# Picky eating in Swedish preschoolers of different weight status: application of two new screening cut-offs

**DOI:** 10.1186/s12966-018-0706-0

**Published:** 2018-08-09

**Authors:** Pernilla Sandvik, Anna Ek, Maria Somaraki, Ulf Hammar, Karin Eli, Paulina Nowicka

**Affiliations:** 1 Department of Food Studies, Nutrition and Dietetics, Uppsala University, Uppsala, Sweden; 2 Unit of Pediatrics, Department of Clinical Science, Intervention and Technology, Karolinska Institutet, Stockholm, Sweden; 3 Institute of Environmental Medicine, Biostatistics, Karolinska Institutet, Stockholm, Sweden; 4 Unit for Biocultural Variation and Obesity, Institute of Social and Cultural Anthropology, University of Oxford, Oxford, UK

**Keywords:** Appetite traits, Eating behavior, Obesity, Parents, Parenting practices, Preschoolers, Screening, Sensory hypersensitivity

## Abstract

**Background:**

Characteristics of picky eaters of different weight status have not been sufficiently investigated. We used two newly developed screening cut-offs for picky eating in the Food fussiness (FF) subscale of the Child Eating Behavior Questionnaire (CEBQ) to investigate the prevalence and characteristics of picky eaters in preschool-aged children with thinness, normal weight, overweight or obesity.

**Methods:**

Data for 1272 preschoolers (mean age 4.9 years) were analyzed. The parent-reported FF subscale ranges from 1 to 5, and two screening cut-offs were applied to classify children as picky eaters (3.0 and 3.33). Structural Equation Modeling was used to study associations with other factors in the CEBQ, the Child Feeding Questionnaire (CFQ) and the Lifestyle Behavior Checklist (LBC). Scores were compared separately for each weight status group.

**Results:**

Nearly half of the children were classified as moderate or severe picky eaters (cut-off 3.0) and 30% as severe (cut-off 3.33). For both cut-offs, prevalence was significantly lower in the obesity group. Still, one-third of children with obesity met the cut-off of 3.0 and 17% met the cut-off of 3.33. While picky eaters displayed similar patterns across weight status groups, some differences emerged. Food responsiveness was lower for picky eaters, but the difference was significant only among children with obesity. Slowness in eating was not as pronounced among picky eaters in the obesity group. In the overweight and obesity groups, parents of picky eaters did not report as high pressure to eat, as compared to the thinness or normal weight groups; in the obesity group, parents of picky eaters also perceived their children’s weight as lower. In all weight status groups, parents of picky eaters were more likely to report their children had too much screen time, complained about physical activity, and expressed negative affect toward food.

**Conclusions:**

Picky eating was less common but still prevalent among children with obesity. Future studies should investigate the potential influence of picky eating on childhood overweight and obesity. Moreover, as children with picky eating display higher emotional sensitivity, further research is needed to understand how to create positive eating environments particularly for children with picky eating and obesity.

**Electronic supplementary material:**

The online version of this article (10.1186/s12966-018-0706-0) contains supplementary material, which is available to authorized users.

## Background

Picky eating refers to a child’s unwillingness to eat familiar foods or try new foods, with negative impacts on children and parents in their daily activities [1]. Studies have reported picky eating in 5 to 60% of children; this wide span is due to differences in definitions, measures and study populations [1]. Children classified as picky eaters eat a selective number of foods, which may result in an insufficient intake of nutrients [1]. Picky eaters may also compensate for their limited intake of disliked food with a higher intake of palatable energy-dense food [2]. Evidence regarding the effect of picky eating on children’s current or future weight status is conflicting [3], but points towards a lower weight and reduced risk of obesity in picky eaters [4]. Yet, picky eating also exists in children with overweight and obesity [5, 6]. In a family-based obesity intervention with preschool children, a reduced degree of picky eating was associated with greater weight reduction [7]. This demonstrates that picky eating may play an important role in overweight and obesity. However, the characteristics of picky eaters of different weight status groups have not been investigated sufficiently. Only one previous study has examined characteristics of picky eaters and non-picky eaters with overweight and obesity [8].

Picky eating often starts in the second year of life [1]. Evidence regarding the trajectory of picky eating prevalence is conflicting. Some longitudinal studies have shown a peak in early childhood and a decline to low levels at around 6 years of age, while others have shown increases in picky eating behaviors from age 2 to a peak at age 6 [9–11]. For most, picky eating is a transient behavior, but, for some children, it is a persistent condition [4, 9]. The severity of picky eating varies, ranging from avoiding or not eating some foods, often meat, fruits and vegetables (especially those with bitter tastes), to consuming a very narrow repertoire of foods; in severe cases, children may also experience anxiety when eating with others [1]. Picky eaters are more likely to be highly sensitive, with elevated perceptions of food texture, smell, taste, sound, visual cues, tactility and motion [12, 13]. Children with moderate or severe picky eating demonstrate a higher degree of psychiatric symptoms, including depression and Attention-Deficit/Hyperactivity Disorder (ADHD) [12]. Children with more emotional temperaments have been reported to display more food avoidant eating behaviors. Specifically, negative affectivity, which is characterized by mood instability, angry reactivity and dysregulated negative emotions have been linked to picky eating in cross-sectional and prospective studies [14–16]. More extreme variants of picky eating may meet diagnostic criteria for Avoidant Restrictive Food Intake Disorder (ARFID) [17]. In this study, we use the term picky eating not as a clinical or diagnostic category, but as a description of eating behavior.

Picky eating is strongly influenced by genetics and the environment [4]. Exposure to flavors and textures increases children’s acceptance of different foods, and several exposures are needed for an initially-rejected food to be accepted [18]. Multiple exposures are particularly important for picky eaters who have sensory hypersensitivity [19]. As parents are key in exposing children to foods and in guiding children’s food practices and social eating behaviors, parental practices are important in shaping children’s acceptance or rejection of food items [13, 20, 21]. When coping with picky eating, parents may apply a variety of techniques to increase children’s food repertoires. Pressuring children to eat – for example, by making sure children clear their plates – is one commonly used technique [22, 23]. This can be problematic for children with obesity, as pressuring a child to eat may hinder the child’s development of well-tuned appetite cues [24, 25]. Coping with a child who has both obesity and picky eating therefore presents a double challenge, as it requires even greater sensitivity in feeding. In the one study examining parents’ feeding practices among picky and non-picky eaters (4–8 years, *n* = 203) with overweight or obesity, monitoring was lower for picky eaters while pressure to eat and child control (the extent to which the child determines what to consume and when) was higher [8]. Among picky eaters with overweight and obesity, greater parental restriction was associated with higher fruit and vegetable intake [8].

The parent-reported Child Eating Behavior Questionnaire (CEBQ) [26] is widely used to assess child eating behaviors among preschoolers [27], including picky eating [1]. Among the eight subscales included in the CEBQ, the six-item food fussiness (FF) subscale focuses on neophobia and general picky eating [26]. Recently, Steinsbekk et al. [28] examined the screening efficiency of the FF subscale for picky eating behavior in a population-based study in Norway. In addition to the scale, the researchers used structured psychiatric interviews with parents of nearly 800 children with a mean age of 6.7 years. Based on this analysis, two cut-offs on the FF subscale were selected, one for moderate and severe cases of picky eating and one for severe cases. These cut-off points have yet to be applied in other populations.

In addition to the CEBQ, the Lifestyle Behavior Checklist (LBC) and the Child Feeding Questionnaire (CFQ) have been widely used to assess the behaviors of children with overweight or obesity, as perceived by their parents, and parental responses to these perceived behaviors. The CFQ has also been used to study associations between parental feeding practices and picky eating among community-based samples of children, showing that parents of picky eaters are more likely to use pressure to eat [29]. However, it is unclear if the same applies to children in different weight status groups, specifically children with overweight or obesity. Moreover, to our knowledge, no previous study involving a large sample of children in different weight status groups has explored how picky eating may be related to other child eating and lifestyle related behaviors and parental feeding practices.

## Aim and hypotheses

This study aims to investigate the prevalence and behavioral characteristics of picky eaters among preschool-aged children with thinness, normal weight, overweight and obesity, using the newly developed CEBQ cut-offs for picky eating. We hypothesize that the prevalence of picky eating will be lower in children with overweight and obesity as compared to children with thinness and normal weight. Further, we hypothesize that for all weight status groups, picky eating will be positively associated with the other food avoidance behaviors in the CEBQ, and negatively associated with the food approach behaviors. In addition, we hypothesize that parents’ controlling feeding behavior scores will be higher for the picky eaters in all weight status groups, specifically for the CFQ subscale pressure to eat, but also for CFQ restriction, particularly in the overweight and obesity groups. Moreover, we hypothesize that behaviors related to overweight and obesity in the LBC, such as excessive screen time and complaining about physical activity, will be less pronounced in children who are picky eaters. Likewise, we hypothesize that behaviors related to negative affectivity will be associated with picky eating in all weight status groups. Finally, we hypothesize that certain covariates will influence picky eating scores: child’s age (decrease with older age), gender (higher in boys), and parental education (higher with lower education).

## Methods

### Participants

Data from three samples were pooled to analyse differences in picky eating characteristics among 1272 children with thinness, normal weight, overweight or obesity. For the first subsample, the Swedish Population Registry was used to recruit all mothers of 4-year-olds in Malmö (*n* = 3007); 876 participated (a response rate of 29%) [30]. For the second subsample, five schools and 20 pre-schools in Stockholm County were recruited from areas with low, medium and high prevalence of obesity; the parents of all children were invited to take part, and 431 completed the questionnaires (a response rate of 46%) [31]. Finally, the third subsample represents baseline data provided by 174 parents of children with obesity in Stockholm. These children participated in a randomised controlled trial for childhood obesity treatment (NCT01792531) [32]. Participant recruitment for all three subsamples has been described in detail previously [30–34].

Of the total sample, *n* = 132 individuals were excluded due to missing values for child weight, height, age or gender. In addition, individuals with fully missing questionnaire responses (*n* = 43) or missing values on the CEBQ food fussiness scale (*n* = 34) were excluded. Thus, this analysis includes 1272 cases. In the excluded subsample, higher percentages of parents had a lower educational level (51% compared to 36% in the final sample) and were born outside Sweden (39% compared to 27% in the final sample).

### Classification of children into four weight status groups

For the children in the obesity treatment group, weight and height were measured by healthcare professionals with calibrated instruments. All measurements were repeated three times and mean values were derived. In the other two subsamples, parents reported their children’s heights and weights.

BMI Z scores were calculated according to Cole and Lobstein [35]. Children were identified as having thinness, normal weight, overweight or obesity based on International Obesity Task Force (IOTF) age- and sex-specific BMI cut-offs [35]. These international child BMI cut-offs correspond to the following body mass index (BMI) at 18 years: thinness: BMI < 18.5 kg/m^2^, normal weight: 18.5 - < 25 kg/m^2^, overweight: 25 - < 30 kg/m^2^ and obesity: ≥ 30 kg/m^2^.

### Measurement of picky eating

Parents completed a Swedish validated version of the CEBQ [31, 36]; the CEBQ FF subscale was used to evaluate picky eating. The CEBQ includes 35 items on eating styles related to obesity risk. These items cluster into eight factors, one of which is food fussiness. The FF subscale includes six items related to neophobia and general picky eating. The items are scored on a five-point Likert scale, from “never” to “always” [26]:

- “My child refuses new food at first”
- “My child enjoys tasting new foods”
- “My child enjoys a wide variety of foods”
- “My child is interested in tasting food s/he hasn’t tasted before”
- “My child decides that s/he doesn’t like food, even without tasting it”
- “My child is difficult to please with meals”

The two cut-offs proposed by Steinsbekk et al. [28] were used to classify the children as non-picky eaters or picky eaters. Steinsbekk et al. [28] examined the screening efficiency of the FF scale using the preschool age psychiatric assessment (PAPA) with the aim of providing meaningful cut-off values [37]. Their interview included questions about children’s food preferences and appetite, as displayed during the last three months. Specifically, parents were interviewed about restrictive food consumption and food selectivity that impaired functioning. In the PAPA, moderate pickiness was defined as the child eating only foods he/she likes, while severe pickiness was defined as substantial and comprehensive restriction, leading to separate meals being made for the child. The cut-point maximizing the sum of sensitivity and specificity for the scale was established at 3.00 for both moderate and severe pickiness and 3.33 for severe cases. Steinsbekk et al. [28] suggest that the FF subscale provides a tool for the identification of clinically significant picky eaters, although further assessment may be needed to separate moderate from severe cases.

### Characteristics of picky and non-picky eaters

The remaining seven factors in the CEBQ were used to explore characteristics of picky versus non-picky eaters in different weight status groups. Four factors are related to food approach: Food responsiveness, Emotional overeating, Enjoyment of food, and Desire to drink. Three are related to food avoidance: Satiety responsiveness, Slowness in eating and Emotional undereating [26]. In accordance with the Swedish validation study, one item from the factor Satiety responsiveness, “My child cannot eat a meal if she has had a snack just before”, was excluded from the present analysis [31].

A Swedish validated version of the Child Feeding Questionnaire (CFQ) was also used [30, 38]. The questionnaire includes 28 items scored on a five-point scale; these items load on six factors: Perceived responsibility, Perceived parent weight, Perceived child weight, Parent’s concern about child weight, Restriction, Pressure to eat and Monitoring. Following the Swedish validation study, two items were excluded: “I offer sweets (candy, ice cream, cake, pastries) as reward for good behavior” and “I offer my child her favorite foods in exchange for good behavior” [30].

The second and third subsample also completed the Lifestyle Behavior Checklist (LBC), an instrument designed to assess parents’ perceptions of children’s obesity-related behaviors. It includes 25 individual statements, scored on a scale from 1 (“not at all”) to 7 (“very much”). A Swedish validated version of the LBC was used; this version consists of a five-factor structure: Misbehavior in relation to food, Overeating, Emotional correlates of being overweight, Physical Activity and Screen Time [39]. The LBC also includes measurements of parents’ self-efficacy in coping with these behaviors (the Confidence scale); however, this was not included in the present analysis. In accordance with the Swedish validation study, six items from the original LBC scale were omitted (items 3, 4, 7, 13, 23, 24) and two items from Physical Activity were transferred to Screen Time [39]. Table 1 shows Cronbach’s alpha for the factors in the CEBQ, LBC and CFQ.

**Table 1:** Cronbach’s alpha for latent factors in the Child Eating Behavior Questionnaire, Child Feeding Questionnaire and Lifestyle Behavior Checklist

	Alpha	No items
Child eating behavior questionnaire (CEBQ) Scale 1–5		
Food fussiness	0.88	6
Enjoyment of food	0.87	3
Emotional overeating	0.77	4
Satiety responsiveness^a^	0.73	4
Slowness in eating	0.80	4
Desire to drink	0.84	3
Emotional undereating	0.79	4
Food responsiveness	0.83	5
Child Feeding Questionnaire (CFQ) Scale 1–5		
Perceived responsibility	0.82	3
Perceived parental weight	0.67	4
Perceived child weight	0.74	3
Concern about child weight	0.88	3
Restriction^c^	0.82	6
Pressure to eat	0.68	4
Monitoring	0.77	3
Lifestyle behavior checklist (LBC) Scale 1–7^b^		
Over eating	0.89	9
Physical activity	0.89	3
Emotional correlates of being overweight	0.74	3
Misbehavior in relation to food	0.78	2
Screen time	0.72	2

^a^ In accordance with a previous validation study, one item was excluded from the factor satiety responsivess in the CEBQ [31]. Cronbach’s alpha with all items were 0.73

^b^ Factor structure according to [39]. In accordance with a previous validation, six item from the original LBC scale was omitted (3, 4, 7, 13, 23, 24) and two items were excluded from the factor PA constructing the separate factor ST [39]

^c^ In accordance with a previous validation study, two items were excluded from the factor restriction in the CFQ [30]. Cronbach’s alpha with all items were 0.80

### Covariates

The background questionnaires consisted of sociodemographic and anthropometric questions from established instruments. The following information was obtained: child’s age, gender, height and weight, and the responding parent’s gender, age, height, weight, foreign/non-foreign background and educational attainment (more than 12 years or 12 years and fewer).

### Statistical methods

Structural Equation Modeling (SEM) was applied to examine differences between weight status groups for mean values on all the subscales. To detect whether associations between picky eating and different questionnaire blocks differed depending on weight status group, we used multi-group generalized SEMs (with weight status as group). We used a logit link, assuming questions to be ordinal-scaled. Each block of questions was assumed to have the same latent factor, with loadings estimated from the data. This latent factor was defined as the outcome and picky eating as the explanatory variable. To simplify the models, error variances and factor loadings were constrained to be equal across obesity groups. For non-converging models, error variances for all items were constrained to equality. All analyses were performed both unadjusted and adjusted. The covariates included in the adjusted model were related to both answering parent (BMI, age, gender, education and foreign/non-foreign background) and child (age and gender). The coefficients of all covariates except for picky eating were constrained to be equal across obesity groups. Due to the properties of SEM, children with missing responses on parts of a questionnaire block could still be included in the analyses. However, children with missing information on an entire block were excluded from the analysis of that block. Children with missing values on covariates were excluded from adjusted analyses, but included in unadjusted analyses. These analyses were done using Stata 15. SPSS 22 was used in regression analyses to study potential associations between picky eating and covariates, where picky eating was treated as both a linear and a binary variable (Steinsbekk’s cut-offs).

## Results

A total of 1272 children were included in the analysis; 15% (*n* = 186) had thinness, 64% (*n* = 813) normal weight, 10% (*n* = 130) overweight and 11% (*n* = 143) obesity. The children’s mean age was 4.9 years (SD 0.8, range 3.3-7.9 years); 49% (*n* = 624) were girls. The children’s characteristics divided by weight status group are shown in Table 2.

**Table 2:** Characteristics of each weight status group in the study sample

	Total n^a^	Thinness (*n* = 186)	Normal weight (*n* = 813)	Over-weight (*n* = 130)	Obesity (*n* = 143)
		Percentage			
Child gender	1272				
Male		53	52	42	49
Female		47	48	58	51
Parent gender	1272				
Male		5	6	8	16
Female		95	94	92	84
Parent university education	1262	58	69	67	43
Parent born in Sweden	1262	67	79	66	57
		Mean (SD)			
Child age	1272	4.8 (0.8)	4.9 (0.8)	4.9 (0.8)	5.1 (0.8)
Parent age	1262	36.2 (4.9)	37.1 (5.0)	36.9 (5.2)	37.4 (6.2)
Child BMI	1272	13.6 (0.6)	15.6 (0.8)	18.3 (0.6)	21.4 (1.7)
Child BMI Z	1272	−1.7 (0.6)	0.1 (0.6)	1.7 (0.3)	3.0 (0.5)
Parent BMI	1196	23.1 (3.1)	23.8 (3.7)	25.3 (5.2)	28.0 (5.7)

^a^Due to missing values, total n does not always add up to the total sample size of *n* = 1272

For each weight status group, the mean scores for the factors in the CEBQ, CFQ and the LBC are shown in Table 3. SEM showed that eating behaviors differed between the weight status groups; children with obesity displayed significantly higher scores for most factors. However, no differences between weight status groups were identified for the CFQ factors *perceived responsibility* and *monitoring*.

**Table 3:** Mean (SD) values for Child Eating Behavior Questionnaire, Child Feeding Questionnaire and Lifestyle Behavior Checklist among children with thinness, normal weight, overweight and obesity

		Thinness		Normal weight		Overweight		Obesity		Differences between weight groups (SEM-analysis)	
	N	Mean	SD	Mean	SD	Mean	SD	Mean	SD	*P*-value	*P*-value adj*
Child Eating Behavior Questionnaire											
Food fussiness	1272	2.9^a^	0.8	2.9^a^	0.8	2.9^a^	0.8	2.6^b^	0.7	0.001	< 0.001
Enjoyment of food	1257	3.2^a^	0.7	3.3^b^	0.7	3.4^b^	0.7	4.0^c^	0.7	< 0.001	< 0.001
Emotional overeating	1248	1.5^a^	0.6	1.4^a^	0.5	1.5^a^	0.5	2.0^b^	0.9	< 0.001	< 0.001
Satiety responsiveness	1244	3.5^a^	0.6	3.3^b^	0.6	3.0^c^	0.6	2.5^d^	0.7	< 0.001	< 0.001
Slowness in eating	1266	3.3^a^	0.8	3.0^b^	0.8	2.8^b^	0.8	2.3^c^	0.8	< 0.001	< 0.001
Desire to drink	1260	2.3^a^	1.0	2.0^b^	0.8	2.3^a^	1.0	2.6^c^	1.0	< 0.001	< 0.001
Emotional undereating	1253	3.1^a^	0.9	2.9^a^	0.9	2.9^a^	0.9	2.5^b^	0.8	< 0.001	< 0.001
Food responsiveness	1258	1.6^a^	0.5	1.7^a^	0.5	2.0^b^	0.8	2.9^c^	1.0	< 0.001	< 0.001
Child Feeding Questionnaire											
Perceived responsibility	1252	4.0	0.7	3.9	0.7	3.9	0.7	4.0	0.8	0.050	.471
Perceived parental weight	1237	3.0^a^	0.4	3.0^b^	0.4	3.1^c^	0.3	3.4^d^	0.5	< 0.001	.440
Perceived child weight	1256	2.7^a^	0.5	3.0^b^	0.4	3.1^c^	0.4	3.5^d^	0.5	< 0.001	< 0.001
Concern	1254	1.3^a^	0.7	1.2^a^	0.6	1.8^b^	1.0	3.3^c^	1.2	< 0.001	< 0.001
Restriction	1225	2.4^a^	0.9	2.5^a^	0.9	3.1^b^	1.0	3.6^c^	0.9	< 0.001	< 0.001
Pressure to eat	1253	3.1^a^	0.9	2.8^b^	0.9	2.6^c^	0.9	2.3^d^	0.9	< 0.001	< 0.001
Monitoring	1261	3.8	0.9	3.8	0.9	4.0	0.7	4.0	0.8	0.610	0.181
Lifestyle Behavior Checklist											
Overeating	501	1.4^a^	0.4	1.4^a^	0.4	2.1^b^	0.9	3.1^c^	1.3	< 0.001	< 0.001
Physical activity	503	1.7^ab^	1.0	1.6^a^	0.9	2.1^b^	1.4	2.5^c^	1.6	< 0.001	< 0.001
Emotional correlates of being overweight	505	1.1^ab^	0.3	1.1^a^	0.3	1.3^b^	0.5	2.0^c^	1.3	< 0.001	< 0.001
Screen time	508	3.0^ac^	1.6	2.5^b^	1.3	2.9^ab^	1.6	3.5^c^	1.5	< 0.001	0.032
Misbehavior in relation to food	505	1.5^ab^	0.8	1.4^a^	0.7	1.9^bc^	1.4	2.3^c^	1.7	< 0.001	< 0.001

*SEM* Structural Equation modelling

^a-d^Different superscript letters indicate significant differences for a specific subscale between weight status groups in the unadjusted model

^*^Adjusted for parental BMI, age, gender, education, foreign background, child age and child gender

### Prevalence of picky eating

The mean total value of the FF-scale was 2.84 (95% CI: 2.80–2.88). Mean FF score was significantly lower among children with obesity (mean 2.59, 95% CI 2.47–2.71) compared to children with thinness (2.88, 95% CI 2.77–3.00), normal weight (2.86, 95% CI 2.81–2.92) and overweight (2.91, 95% CI 2.77–3.06). No covariate showed a significant association with food fussiness neither as a linear variable nor when using the cut-offs.

Using Steinsbekk’s cut-off of 3.00 (including moderate and severe picky eating), 47% (*n* = 592) of children were classified as picky eaters. When divided by weight status group, the percentages of picky eaters were 52% (*n* = 96) of children with thinness, 47% (*n* = 381) of children with normal weight, 49% (*n* = 63) of children with overweight and 36% (*n* = 52) of children with obesity.

Using Steinsbekk’s cut-off of 3.33, the percentage of children classified as severe picky eaters was 30% (*n* = 387), including 32% (*n* = 60) of the thinness group, 32% (*n* = 258) of the normal weight group, 35% (*n* = 45) of the overweight group and 17% (*n* = 24) of the obesity group. There were no significant differences in picky eating between the children with obesity drawn from the clinical sample (*n* = 113) and the non-clinical samples (*n* = 30) (*p*-value 0.180).

### Associations between picky eating and parental and child behaviors in different weight status groups

We used Steinsbekk’s cut-offs to examine the eating and obesity-related behaviors of picky eaters in different weight status groups, as perceived by parents, alongside parental feeding practices. Table 4 shows the direction of associations between picky eating (cut-off 3.00) and studied behaviors, divided by weight status group, using the adjusted model (the unadjusted model table appears as Additional file 1).

**Table 4:** Adjusted models comparing picky and non-picky eaters in specific weight groups, using the cut-off 3.0 in the Food Fussiness subscale of the Child Eating Behavior Questionnaire

		Thinness		Normal weight		Overweight		Obesity		Overall P**
	n	Coeff.	*P*-value*	Coeff.	*P*-value*	Coeff.	*P*-value*	Coeff.	*P*-value*	
Child Eating Behavior Questionnaire										
Enjoyment of food	1183	**−2.924**	< 0.001	**−3.089**	< 0.001	**−2.655**	< 0.001	**−2.501**	< 0.001	0.743
Emotional overeating	1183	−0.149	0.710	0.114	0.563	0.374	0.424	0.574	0.232	0.661
Satiety responsiveness	1183	**1.217**	< 0.001	**1.154**	< 0.001	**1.201**	< 0.001	**0.758**	0.021	0.676
Slowness in eating	1183	**1.078**	0.002	**1.080**	< 0.001	**1.109**	0.024	0.835	0.066	0.966
Desire to drink	1183	−0.284	0.258	**0.432**	0.037	0.400	0.530	−0.870	0.086	0.074
Emotional undereating	1183	0.455	0.245	**1.333**	< 0.001	**2.327**	< 0.001	**1.414**	0.004	**0.046**
Food responsiveness	1183	−0.253	0.301	−0.251	0.067	−0.598	0.190	**−1.236**	0.006	0.183
Child Feeding Questionnaire										
Perceived responsibility	1176	0.161	0.824	−0.331	0.259	−0.049	0.213	−0.624	0.472	0.752
Perceived parental weight	1178	−0.118	0.665	−0.123	0.302	−0.173	0.601	−0.178	0.501	0.997
Perceived child weight	1177	−0.208	0.696	−0.478	0.140	0.129	0.820	**−0.611**	0.042	0.693
Concern about child weight	1175	−0.455	0.359	0.036	0.903	−0.446	0.424	−0.526	0.294	0.698
Restriction	1177	−0.545	0.172	0.272	0.149	0.036	0.936	−0.232	0.618	0.265
Pressure to eat	1177	**0.626**	0.029	**0.941**	< 0.001	0.324	0.285	0.318	0.229	0.070
Monitoring	1176	**−1.092**	0.023	−0.060	0.781	0.106	0.837	−1.095	0.059	0.090
Lifestyle Behavior Checklist										
Overeating	497	0.377	0.250	0.270	0.053	−0.229	0.594	− 0.198	0.539	0.374
Physical activity	494	**2.686**	0.022	**1.426**	0.002	**3.184**	0.016	0.726	0.313	0.348
Emotional correlates of overweight^a^	495	− 1.557	0.324	**0.946**	0.021	**1.362**	0.045	0.698	0.124	0.362
Screen Time	494	1.561	0.145	**0.714**	0.037	1.093	0.293	**0.865**	0.018	0.882
Misbehavior in relation to food^a^	497	**1.917**	0.002	**1.400**	0.001	1.838	0.110	0.380	0.658	0.538

Structural Equation Model is adjusted for parental BMI, age, gender, education, foreign background, child age and child gender

All boldface coefficients correspond to a significant p-value (<0.05)

^a^ not adjusted for parental age, due to non-converging model

*p < .05 indicate significant difference between picky and non-picky eaters for subscale in the specific weight status group

**p < .05 indicate significant differences among the four weight status groups in the associations between picky eating and a specific subscale

Overall, the most distinct differences between picky and non-picky eaters in all weight groups were found in eating behaviors (CEBQ) and obesity-related behaviors (LBC). For parental feeding practices (CFQ), the differences between the parents of picky and non-picky eaters were minor.

Picky eaters in all weight status groups displayed a lower enjoyment of food and higher scores of emotional undereating. Notably, contrary to the other weight groups, picky eaters with obesity did not display a higher degree of slowness in eating; however, they did display a lower degree of food responsiveness compared to other children with obesity. Also contrary to the other weight groups, picky eaters of normal weight showed a higher desire to drink compared to the non-picky eaters in their weight group.

Pressure to eat was positively associated with picky eating among children with thinness or normal weight, but not among children with overweight or obesity. In the obesity group, picky eating was associated with parental perceptions of child weight: the weight of children, as perceived by parents, was lower for picky eaters than for non-picky eaters. Among parents of children with obesity and picky eating, 35% perceived their child to have normal body weight as compared to 9% in the non-picky group. Based on this finding, we conducted further analysis to determine whether the picky eaters and the non-picky eaters in the obesity group differed in body weight. We found that the children classified as picky eaters had significantly lower weight (BMI-Z 2.9) than the non-picky eaters (BMI-Z, 3.1) in the obesity group (*p* = 0.038).

Results from the LBC showed that, in all weight status groups, parents of picky eaters were more likely to report that their children complained about physical activity, compared to the parents of non-picky eaters; the difference was not significant in the obesity group. For picky eaters with obesity, having too much screen time was more common compared to non-picky eaters with obesity. Picky eaters in the normal weight and the overweight groups displayed higher scores for the factor Emotional correlates of being overweight, which included negative feelings over being teased, being overweight and clothes not fitting.

Table 5 provides the same analysis using the higher cut-off of 3.33. While most patterns were the same, a few differences emerged. For the obesity group, slowness in eating differed between the picky and non-picky eaters. Also in the obesity group, while the difference between perceptions of the child’s body weight among parents of picky eaters and parents of non-picky eaters was no longer significant, it did trend in the same direction. The higher degree of desire to drink among picky eaters in the normal weight group was no longer present.

**Table 5:** Adjusted models comparing picky and non-picky eaters in specific weight groups, using the cut-off 3.3 in the Food Fussiness subscale of the Child Eating Behavior Questionnaire

		Thinness		Normal weight		Overweight		Obesity		Overall P**
	n	Coeff.	*P*-value*	Coeff.	*P*-value*	Coeff.	*P*-value*	Coeff.	*P*-value*	
Child Eating Behavior Questionnaire										
Enjoyment of food	1183	**−3.031**	< 0.001	**−3.261**	< 0.001	**− 2.979**	< 0.001	**− 2.793**	0.001	0.905
Emotional overeating	1183	0.580	0.169	−0.057	0.787	−0.293	0.547	0.693	0.269	0.341
Satiety responsiveness	1183	**0.865**	0.001	**1.257**	< 0.001	**1.239**	< 0.001	**0.865**	0.034	0.451
Slowness in eating	1183	**1.169**	0.001	**1.051**	< 0.001	**1.591**	< 0.001	**1.260**	0.035	0.789
Desire to drink	1183	−0.009	0.984	0.224	0.314	0.525	0.427	−0.801	0.237	0.472
Emotional undereating	1183	**0.842**	0.042	**1.424**	< 0.001	**1.368**	0.026	**1.605**	0.012	0.621
Food responsiveness	1183	−0.170	0.515	**−0.422**	0.004	**−1.152**	0.015	−0.889	0.138	0.267
Child Feeding Questionnaire										
Perceived responsibility^a^	1182	0.002	0.997	−0.125	0.673	−0.502	0.540	−1.529	0.161	0.647
Perceived parental weight	1178	−0.012	0.996	0.037	0.771	0.345	0.326	−0.084	0.808	0.819
Perceive child weight	1177	−0.354	0.530	−0.450	0.191	0.329	0.578	−0.421	0.289	0.714
Concern about child weight	1175	0.009	0.986	−0.007	0.982	−0.046	0.937	−0.234	0.727	0.991
Restriction^a^	1183	−0.342	0.409	0.251	0.207	0.016	0.973	0.009	0.988	0.616
Pressure to eat	1177	**0.663**	0.029	**0.932**	< 0.001	0.364	0.247	**0.890**	0.007	0.383
Monitoring	1176	−0.447	0.382	−0.083	0.720	0.188	0.728	−0.728	0.343	0.713
Lifestyle Behavior Checklist										
Overeating	497	0.230	0.486	0.240	0.103	−0.535	0.227	−0.307	0.486	0.266
Physical activity	494	**2.957**	0.017	**1.955**	< 0.001	**3.048**	0.028	0.465	0.634	0.357
Emotional correlates of overweight	494	−0.377	0.798	**0.800**	0.036	1.128	0.053	0.324	0.611	0.630
Screen Time	494	**2.349**	0.034	0.450	0.210	1.337	0.207	**0.465**	0.007	0.226
Misbehavior in relation to food^b^	510	**2.262**	0.002	**1.573**	< 0.001	2.049	0.057	1.772	0.113	0.869

Structural Equation Model is adjusted for parental BMI, age, gender, education, foreign background, child age and child gender

All boldface coefficients correspond to a significant p-value (<0.05)

^a^ not adjusted for parental age, due to non-converging model

^b^ unadjusted model, adjusted model did not converge

*p < .05 indicate significant difference between picky and non-picky eaters for subscale in the specific weight status group

**p < .05 indicate significant differences among the four weight groups in the associations between picky eating and a specific subscale

## Discussion

In a large sample of preschoolers divided into four weight status groups, picky eating was prevalent according to two new screening cut-offs for moderate and severe picky eating. Nearly half of the children with thinness, normal weight and overweight were classified as having moderate or severe picky eating. The prevalence among children with obesity was lower; still, one-third were classified as picky eaters. Picky eaters in different weight status groups displayed similar patterns with regards to the other subscales of the CEBQ, the CFQ and the LBC; however, some differences emerged. CEBQ Food responsiveness was lower for picky eaters, but the difference was significant only among children with obesity. In contrast, CEBQ slowness in eating was not as pronounced among picky eaters in the obesity group. Parents of picky eaters in the overweight and obesity groups did not report more CFQ pressure to eat as compared to the underweight and normal weight group. Additionally, parents of children with obesity and picky eating perceived their children as having a lower weight in the CFQ, compared to parents of non-picky eaters with obesity. Across the sample, parents of picky eaters were more likely to report in the LBC that their children complained about physical activity, and that their children had too much screen time.

Comprehensive prevalence studies on picky eating are needed. The lack of standardized measurements, which has resulted in a wide span of estimated prevalence rates, from five to 60%, makes our findings difficult to compare. Few studies have investigated picky eating in different weight status groups. In a sample of 1225 Portuguese children with a mean age of eight years, picky eating was identified through parents’ responses (“yes” or “no”) to the item “My child eats everything”, and was found in 26% of the normal weight, 14% of the overweight and 7% of the obesity group [5]. The above findings are in line with the Dutch Generation R study where the degree of pickiness among four-year-olds (*n* = 4987) also decreased with increased weight [40]. Like our study, the Dutch study used the FF subscale; however, in our study, only the obesity group had a lower prevalence of picky eating compared to the other weight groups. In contrast, Finistrella et al. [6], in a small study of Italian preschoolers (*n* = 127, mean age 4.7 years), found that children with overweight and obesity were somewhat more picky compared to their normal-weight peers. Here, pickiness was evaluated by parents responding to three items: “My child’s diet consists of only a few foods”, “My child is unwilling to eat many of the foods that our family eats at mealtimes” and “My child is fussy or picky about what she eats”.

Picky eating is linked with two dispositional qualities in children: negative affectivity and sensory sensitivity [13]. Both were included by Steinsbekk et al. [13] in their etiological model of picky eating. In our population, picky eaters in all weight status groups displayed more behaviors related to negative affectivity, similar to findings by Jacobi et al. [14] and Haycraft et al. [15]. Negative affectivity was measured through the CEBQ subscale Emotional undereating and the LBC subscale Emotional correlates of being overweight. It is possible that picky eaters are more likely to display negative affect relating to food and weight, or that their parents are more likely to report such responses. Picky eaters in all weight status groups also displayed a lower degree of positive emotions related to food, as measured by the CEBQ subscale Enjoyment of food, which includes items such as looking forward to meals, loving food and being interested in food. Hence, picky eaters seem to react to food with more ambivalent or negative emotions, in line with previous research [16]. Picky eating has been connected to increased anxiety over foods, but also to elevated levels of general anxiety [41]. High sensory sensitivity has been linked with picky eating at the age of four, and with a higher risk of persistent picky eating two years later [13]. Picky eaters might therefore represent a highly sensitive group of children. Supporting parents in reacting appropriately is important for the development of these children’s eating habits, since responding by increased pressure to eat may increase picky eating [42].

Parents of picky eaters with thinness and normal weight reported applying greater pressure to eat. They also reported their children displayed a higher degree of misbehavior in relation to food, such as yelling and throwing tantrums about food, which could be a reaction to pressure to eat. Contrary to our hypothesis and to previous research, pressure to eat was not as pronounced among parents of picky eaters in the overweight and obesity group [8]. This is positive, because pressuring children to eat may negatively affect their own ability to regulate food intake [24, 25]. Indeed, in the etiological model proposed by Steinsbekk et al. [13], parent-child interaction was the third component; this was confirmed in a prospective study that found that responsive parenting (parental structuring) reduced the risk of picky eating two years later.

Contrary to our hypothesis, parents of picky eaters were more likely to report their children complained about being physically active and had longer screen time. This could indicate a lower degree of physical activity among picky eaters, which, to our knowledge, has not been previously shown. The finding is also interesting in relation to previous research that found an association between picky eating and ADHD [12]. A recent study, however, confirmed an association only with the impulsivity component of ADHD and not the inattention or hyperactivity components [43]. Problematic screen time behaviors among picky eaters match clinical experiences from our group, as we have encountered parents who say that putting a screen in front of their children is the only way to get them to eat. We relate this finding to previous studies showing that picky eaters have enhanced sensory sensitivity. Zucker et al. [12] argue that this enhanced sensitivity makes it challenging to regulate emotions or modulate attention. Screens could perhaps distract children from the sensory qualities of food. This also relates to previous research that found that children who are perceived as fussier in general have more TV exposure [44].

The present study shows that picky eating is prevalent in preschoolers with overweight and obesity. As Hayes et al. [7] recently showed, picky eating may play a role in obesity treatment and should therefore be addressed by healthcare professionals. Parents of picky eaters in the obesity group perceived their child’s weight to be lower and 35% perceived their child to have normal weight; these parents also showed a non-significant trend towards not being as concerned about the child’s weight. This could indicate that picky eating is perceived as more problematic than the child’s weight, since it affects everyday life. Notably, picky eaters with obesity had significantly lower scores on several obesity-related eating behaviors, displaying less enjoyment of food, higher satiety responses, more emotional undereating and less food responsiveness. They did, however, score higher on other obesity-related lifestyle behaviors, such as having more screen time. Perhaps the genetic component of obesity plays a greater role for these children than an obesity-related behavior pattern [4, 45]. In this case, physical activity may be even more important.

To our knowledge, this is the first study that uses the two recently proposed cut-offs for picky eating. Our study identified several behavioral and psychosocial differences between picky eaters and non-picky eaters. Our results call into question the clinical usefulness of the cut-offs in our population, given that one-third of the children were classified as severe picky eaters. Moreover, the cut-off of 3.00 also includes the option ‘sometimes’ on the FF scale; occasional picky eating might be normal for young children, and the mean age of our sample was two years younger than the population studied by Steinsbekk et al., which might have increased the proportion of children classified as picky eaters [28]. Nevertheless, it is difficult to use the cut-offs to identify those children who may need clinical support if so many children are classified as picky eaters.

Our study had several limitations. The data were limited by the questionnaires’ reliance on parental perceptions of behaviors, meaning that parents could report misperceived behaviors [46]. Another limitation was the reliance on self-reported height and weight data in the population and school-based samples, although this was the most feasible method given these subsamples’ large sizes. Both overestimation and underestimation are possible, especially for children with obesity [47]; however, the clinical group, which included most children with obesity, was measured by healthcare professionals. Notably, young children in Sweden are invited to participate in annual check-ups at their child healthcare center, where height and weight are measured by an experienced nurse; moreover, school nurses measure the heights and weights of all six-year-olds. Both practices increase parents’ ability to report their child’s weight and height accurately. Further, the generalizability of the results is limited by in the study’s location in Sweden; approximately 60% of the responding parents had a university degree and only 33% had a non-Swedish background. Finally, the cross-sectional nature of the study did not allow for causal inferences.

Future research should further develop valid measurements of picky eating, taking into consideration the various ways in which picky eating may be expressed and perceived. Given the few studies in this field, research is warranted among both younger and older children. Moreover, clinical and health education strategies to support parents in handling picky eating should be developed, particularly focusing on parents who experience the double challenge of picky eating and obesity. As the current study indicates that children with picky eating may have enhanced sensory and emotional sensitivity, their parents need additional support to create a positive eating environment.

## Conclusions

Using the newly developed cut-offs for picky eating, we examined the characteristics of picky and non-picky eaters with regard to parents’ perceptions of eating behaviors, their own parental feeding practices and the children’s obesity-related behaviors. Nearly half of the children in the total sample and one third of the children with obesity were classified as having moderate or severe picky eating. A higher proportion of parents of picky eaters with obesity perceived their child to have normal weight, compared to parents of non-picky eaters with obesity. Picky eaters with obesity displayed a lower degree of obesity-related eating behaviors, but higher degrees of sedentary behaviors. Future studies should investigate the potential influence of picky eating on childhood overweight and obesity. Moreover, as children with picky eating in all weight status groups display higher emotional sensitivity and negative affectivity, further research is needed to understand how to create positive eating environments for children with picky eating. More studies are also needed to ascertain how parents, especially of children with obesity and picky eating, can create a positive environment for both healthy eating and physical activity.

## Additional file

Additional file 1: **Table S1.** Unadjusted models comparing picky and non-picky eaters in specific weight groups, using the cut-off 3.0. **Table S2.** Undjusted models comparing picky and non-picky eaters in specific weight groups, using the cut-off 3.3. (DOCX 29 kb)

## Abbreviations

ADHD: Attention-deficit/hyperactivity disorder
ARFID: Avoidant restrictive food intake disorder
BMI: Body Mass Index
CEBQ: Child eating behavior questionnaire
CFQ: Child feeding questionnaire
FF: Food fussiness
LBC: Lifestyle behavior checklist
