# Supplementary material for: Picky eating in Swedish preschoolers of different weight status: application of two new screening cut-offs
Source: Int J Behav Nutr Phys Act. 2018 Aug 9;15:74. doi: 10.1186/s12966-018-0706-0 (PMC6085619; doi:10.1186/s12966-018-0706-0)
Supplement: Supplementary file 1 — Table S1. Unadjusted models comparing picky and non-picky eaters in specific weight groups, using the cut-off 3.0. Table S2. Undjusted models comparing picky and non-picky eaters in specific weight groups, using the cut-off 3.3. (DOCX 29 kb) [file 12966_2018_706_MOESM1_ESM.docx]

Additional file 1

**Table S1.** Unadjusted models comparing picky and non-picky eaters in specific weight groups, using the cut-off 3.0.

|  |  | Thinness |  | Normal weight |  | Overweight |  | Obesity |  | Overall P** |
| --- | --- | --- | --- | --- | --- | --- | --- | --- | --- | --- |
|  | n | Coeff. | P-value* | Coeff. | P-value* | Coeff. | P-value* | Coeff. | P-value* |  |
| Child Eating Behavior Questionnaire |  |  |  |  |  |  |  |  |  |  |
| Enjoyment of food | 1272 | **-0.285** | <0.001 | **-3.087** | <0.001 | **-2.421** | <0.001 | **-2.432** | <0.001 | 0.530 |
| Emotional overeating | 1272 | 0.079 | 0.816 | 0.043 | 0.788 | 0.201 | 0.601 | 0.702 | 0.061 | 0.485 |
| Satiety responsiveness | 1272 | **1.260** | <0.001 | **1.092** | <0.001 | **1.081** | 0.001 | **0.815** | 0.008 | 0.692 |
| Slowness in eating | 1272 | **1.088** | 0.001 | **1.091** | <0.001 | 0.865 | 0.054 | 0.876 | 0.054 | 0.946 |
| Desire to drink | 1272 | -0.298 | 0.385 | 0.338 | 0.016 | 0.132 | 0.753 | -0.354 | 0.241 | 0.155 |
| Emotional undereating | 1272 | -0.390 | 0.333 | **1.225** | <0.001 | **2.313** | <0.001 | **1.302** | 0.004 | **0.039** |
| Food responsiveness | 1272 | -0.008 | 0.973 | **-0.257** | 0.050 | -0.537 | 0.178 | **1.026** | 0.005 | 0.123 |
| Child Feeding Questionnaire |  |  |  |  |  |  |  |  |  |  |
| Perceived responsibility | 1260 | 0.346 | 0.563 | -0.361 | 0.194 | -0.139 | 0.075 | -0.701 | 0.391 | 0.378 |
| Perceived parental weight | 1262 | -0.036 | 0.905 | -0.160 | 0.281 | -0.133 | 0.661 | -0.265 | 0.395 | 0.962 |
| Perceived child weight | 1261 | -0.510 | 0.309 | -0.416 | 0.175 | 0.155 | 0.782 | **-0.664** | 0.035 | 0.653 |
| Concern about child weight | 1261 | -0.027 | 0.963 | 0.016 | 0.959 | -0.411 | 0.473 | -0.449 | 0.329 | 0.835 |
| Restriction | 1263 | -0.279 | 0.502 | 0.308 | 0.106 | -0.149 | 0.742 | -0.148 | 0.719 | 0.442 |
| Pressure to eat | 1263 | **0.301** | <0.001 | **0.341** | <0.001 | 0.127 | 0.096 | 0.095 | 0.181 | **0.007** |
| Monitoring | 1262 | **-0.864** | 0.044 | -0.112 | 0.591 | 0.248 | 0.598 | **-1.162** | 0.038 | 0.100 |
| Lifestyle Behavior Checklist |  |  |  |  |  |  |  |  |  |  |
| Overeating | 511 | 0.276 | 0.383 | **0.293** | 0.036 | -0.271 | 0.515 | -0.146 | 0.642 | 0.390 |
| Physical activity | 508 | 2.341 | 0.051 | **1.615** | 0.002 | **3.316** | 0.023 | 0.587 | 0.431 | 0.348 |
| Emotional correlates of overweight | 508 | -0.617 | 0.598 | **0.848** | 0.038 | **1.613** | 0.022 | 0.781 | 0.131 | 0.410 |
| Screen Time | 508 | 1.492 | 0.179 | 0.608 | 0.076 | 0.546 | 0.114 | **0.760** | 0.045 | 0.728 |
| Misbehavior in relation to food*** | 510 | **1.832** | 0.017 | **1.447** | <0.001 | 1.367 | 0.185 | 0.567 | 0.508 | 0.712 |

Structural Equation Model is adjusted for parental weight, age, gender, education, foreign background, child age and child gender.
* p<.05 indicated difference between picky and non-picky eaters in the specific weight group.

** p<.05 indicate differences among the four weight groups in the associations between picky eating and a specific subscale

**Table S2.** Undjusted models comparing picky and non-picky eaters in specific weight groups, using the cut-off 3.3.

|  |  | Thinness |  | Normal weight |  | Overweight |  | Obesity |  | Overall P** |
| --- | --- | --- | --- | --- | --- | --- | --- | --- | --- | --- |
|  | n | Coeff. | P-value* | Coeff. | P-value* | Coeff. | P-value* | Coeff. | P-value* |  |
| Child Eating Behavior Questionnaire |  |  |  |  |  |  |  |  |  |  |
| Enjoyment of food | 1272 | **-3.307** | <0.001 | **-3.236** | <0.001 | **-2.691** | <0.001 | **-2.643** | 0.001 | 0.724 |
| Emotional overeating | 1272 | 0.420 | 0.245 | -0.078 | 0.653 | -0.246 | 0.543 | 0.741 | 0.122 | 0.280 |
| Satiety responsiveness | 1272 | **0.858** | <0.001 | **1.140** | <0.001 | **1.088** | 0.001 | **0.825** | 0.029 | 0.636 |
| Slowness in eating | 1272 | **1.217** | 0.001 | **0.989** | <0.001 | **1.192** | 0.011 | **1.213** | 0.040 | 0.928 |
| Desire to drink | 1272 | -0.140 | 0.698 | 0.176 | 0.237 | 0.088 | 0.829 | -0.011 | 0.977 | 0.834 |
| Emotional undereating | 1272 | 0.661 | 0.122 | **1.246** | <0.001 | **1.473** | 0.013 | **1.290** | 0.027 | 0.601 |
| Food responsiveness | 1272 | 0.182 | 0.502 | **-0.389** | 0.006 | **-1.069** | 0.011 | -0.743 | 0.118 | 0.295 |
| Child Feeding Questionnaire |  |  |  |  |  |  |  |  |  |  |
| Perceived responsibility | 1260 | 0.559 | 0.383 | -0.166 | 0.579 | -1.280 | 0.123 | -2.028 | 0.066 | 0.147 |
| Perceived parental weight | 1262 | -0.026 | 0.934 | 0.052 | 0.732 | 0.096 | 0.760 | -0.029 | 0.943 | 0.990 |
| Perceived child weight | 1261 | -0.804 | 0.130 | -0.361 | 0.267 | 0.321 | 0.586 | -0.379 | 0.358 | 0.574 |
| Concern about child weight | 1261 | 0.380 | 0.536 | 0.000 | 1.000 | -0.377 | 0.352 | -0.073 | 0.904 | 0.863 |
| Restriction | 1263 | -0.206 | 0.641 | 0.218 | 0.285 | -0.292 | 0.540 | -0.040 | 0.940 | 0.673 |
| Pressure to eat | 1263 | **0.280** | 0.004 | **0.340** | <0.001 | 0.130 | 0.099 | **0.212** | 0.025 | 0.113 |
| Monitoring | 1262 | -0.679 | 0.136 | -0.112 | 0.616 | 0.221 | 0.656 | -0.729 | 0.321 | 0.478 |
| Lifestyle Behavior Checklist |  |  |  |  |  |  |  |  |  |  |
| Overeating | 511 | 0.141 | 0.658 | 0.277 | 0.060 | -0.549 | 0.200 | -0.065 | 0.877 | 0.293 |
| Physical activity | 508 | **2.553** | 0.041 | **2.213** | <0.001 | **3.410** | 0.025 | 0.760 | 0.443 | 0.483 |
| Emotional correlates of overweight | 508 | 0.323 | 0.781 | **0.828** | 0.037 | 1.059 | 0.122 | 0.675 | 0.320 | 0.950 |
| Screen Time | 508 | 2.010 | 0.079 | 0.421 | 0.244 | 1.808 | 0.069 | **1.452** | 0.004 | 0.187 |
| Misbehavior in relation to food | 510 | **2.262** | 0.002 | **1.573** | <0.001 | 2.049 | 0.057 | 1.772 | 0.113 | 0.869 |

Structural Equation Model is adjusted for parental weight, age, gender, education, foreign background, child age and child gender.
* p<.05 indicated difference between picky and non-picky eaters in the specific weight group.

** p<.05 indicate differences among the four weight groups in the associations between picky eating and a specific subscale
